# Mapping the research landscape of oral appliances in obstructive sleep apnea: a bibliometric analysis of trends, influential publications, and emerging areas

**DOI:** 10.1038/s41405-025-00305-z

**Published:** 2025-03-31

**Authors:** Gowri Sivaramakrishnan, Kannan Sridharan

**Affiliations:** 1 Bahrain Defence Force Royal Medical Services, Riffa, Bahrain; 2 Department of Pharmacology and Therapeutics, College of Medicine and Health Sciences, Arabian Gulf University, Manama, Bahrain

**Keywords:** Removable prosthodontics, Removable prosthodontics

## Abstract

**Background:**

Oral appliances (OAs) are widely used in the management of obstructive sleep apnea (OSA), yet a comprehensive understanding of the research landscape in this field is lacking. This study aims to map the global research trends, influential publications, leading researchers, and emerging areas of interest related to OAs for OSA.

**Methodology:**

Data were retrieved from the Scopus. The search included terms related to OSA and OA. Articles were screened using Rayyan software. VOS viewer™ and Bibliometrix were used for analysis. Data were visualized through network maps and graphs to identify key authors, research centers, countries, and keyword trends. Co-occurrence of keywords and citation patterns were assessed to understand the research dynamics.

**Results:**

Out of 1370 initially retrieved articles, 753 were selected for final analysis, revealing a marked increase in scientific output in recent years. The study identified approximately 2400 researchers, with notable work from Cistulli P.A., Vanderveken O.M., and Lowe A.A., who formed key clusters. Major research hubs included The University of British Columbia, The University of Sydney, and Royal North Shore Hospital. The USA and Japan led in citations and publications. Global collaboration patterns were evident, showing contributions from various countries. Keywords like “obstructive sleep apnea,” “mandibular advancement device,” and “oral appliance” were frequently used, while emerging trends highlighted gaps in research related to tongue retaining and hybrid appliances. The top 20 cited documents from 1995–2020 encompassed reviews, clinical practice guidelines, and randomized trials, with the “Sleep” journal being the most cited source.

**Conclusion:**

This bibliometric analysis provides a detailed overview of the research landscape on OAs for OSA. The study highlights significant trends, influential researchers, and key research centers. It also identifies emerging areas of interest and research gaps, offering guidance for future research to enhance the clinical effectiveness and adoption of OA therapy for OSA.

## Introduction

Obstructive sleep apnea (OSA) is a prevalent sleep disorder characterized by repeated episodes of partial or complete blockage of the upper airway during sleep, leading to pauses in breathing, reduced oxygen saturation, and frequent awakenings. This disruption not only affects sleep quality but also contributes to serious health conditions like hypertension, cardiovascular diseases, and stroke [1].

Among the various treatment options for OSA, continuous positive airway pressure (CPAP) stands out as a well-established, non-invasive therapy. It delivers a constant flow of air through a mask, maintaining positive pressure in the upper airway during sleep [2]. This continuous airflow helps prevent airway collapse, ensuring uninterrupted breathing and proper oxygenation throughout the night. CPAP is typically the recommended first-line treatment for moderate to severe OSA, thanks to its high success rate in enhancing sleep quality, alleviating daytime sleepiness, and reducing long-term health risks, including cardiovascular diseases, hypertension, and stroke [1, 2]. However, despite its proven clinical effectiveness, CPAP therapy often suffers from low patient adherence, primarily due to factors such as discomfort, noise, poor mask fit, and the inconvenience of wearing the device throughout the night [3].

Oral appliances (OA) have emerged as an effective and non-invasive choice, especially for patients with mild to moderate OSA. These custom-made devices are designed to be worn during sleep and function by maintaining an open airway, typically by repositioning the mandible and tongue to prevent airway collapse [4]. The two main types of oral appliances are Mandibular Advancement Devices (MADs) and Tongue-Retaining Devices (TRDs). MADs work by gently advancing the lower jaw forward, which in turn increases the airway space and reduces the likelihood of obstruction, while TRDs keep the tongue in a forward position, preventing it from collapsing into the airway [5]. Oral appliances are often used as an alternative to Continuous Positive Airway Pressure (CPAP) therapy, especially for patients who find CPAP uncomfortable or difficult to tolerate. These appliances are typically fitted by dental professionals trained in sleep medicine, such as prosthodontists. The effectiveness of oral appliances varies depending on the severity of OSA and the specific design of the device, but they offer a comfortable and convenient solution that improves sleep quality and overall health for many patients [6].

Current research trends in the use of oral appliances for OSA are focused on improving device design, patient compliance, and personalized treatment outcomes. Studies are increasingly investigating the long-term efficacy of MADs compared to traditional treatments like CPAP, as well as their impact on reducing cardiovascular risks associated with OSA [7]. Advances in 3D imaging and digital workflows are also being explored to enhance the precision of OA fabrication and fitting. Additionally, there is growing interest in understanding the genetic and anatomical predictors of oral appliance success, allowing for more tailored therapies based on individual patient characteristics [8]. As the body of research on OAs expands, bibliometric analysis becomes essential to map the evolution of this field, identify influential studies, and highlight emerging research areas. By analyzing citation patterns, co-authorship networks, and keyword trends, bibliometric analysis helps researchers and clinicians stay informed of the latest developments, guide future research, and improve evidence-based decision-making in clinical practice.

The aim of the present study is to conduct a comprehensive bibliometric analysis of the global research landscape on OAs used for the management of OSA, in order to identify key trends, influential publications, prominent researchers, and emerging areas of interest. This analysis will provide valuable insights into the evolution of research in this field, highlight gaps in current knowledge, and guide future research directions to improve the clinical effectiveness and adoption of OA therapy for OSA.

## Methodology

### Search strategy and database

The data analyzed were sourced from the Scopus database. The search was performed in August 2024, without restrictions on language or publication year. The following search strategy was used to perform the search: (TITLE-ABS-KEY (obstructive AND sleep AND apnea) OR TITLE-ABS-KEY (obstructive AND sleep AND apnea AND syndrome) AND TITLE-ABS-KEY (prosthesis) OR TITLE-ABS-KEY (oral AND appliance)).

### Inclusion and exclusion criteria

The search results were uploaded in Rayyan software for screening [9]. Two authors independently screened the articles for inclusion. Any disagreement between the authors was resolved through discussion. Only journal articles including clinical studies, observational studies, cross-sectional studies, case reports, case series and review articles on the use of oral appliances for OSA were included. Commentaries, letters, editorials, opinion and book chapters were excluded.

### Bibliometric analysis

The VOS viewer™ software (version 1.6.18) for Mac and Bibliometrix were utilized for visualizing bibliometric analyses. A CSV file containing citation and bibliographic information, abstracts, and keywords was employed. VOS viewer™ was used to examine publication characteristics, such as identifying the most cited researchers, the most significant affiliations, the most cited countries, countries with the highest publication output, keyword co-occurrences, the most cited sources, notable authors, and highly cited documents. This analysis produced maps and graphs for visualizing and interpreting the data. The items under study were organized into clusters, with each cluster represented on a map and labeled with cluster numbers. For easier interpretation, items were represented as “nodes,” with the connections between them referred to as “edges.” The strength of these connections was represented as “edge weight.” Depending on the analysis performed, different nodes represented various terms, with their sizes reflecting the number of citations, co-citations, and keyword co-occurrences. Nodes and lines within the same cluster were color-coded similarly. Using VOS viewer™, network visualization maps were generated for the most cited researchers, the most cited countries, significant documents, and the timeline of related publications.

Bibliometrix is an open-source software specifically tailored for conducting a wide range of quantitative analyses, particularly emphasizing the visual representation of data. It facilitates the creation of various types of maps and graphs, enabling a clearer understanding of relationships and trends within the data. Additionally, it constructs data matrices for in-depth analyses, including co-citation networks, scientific collaboration patterns, and keyword frequency or association studies. This comprehensive approach aids researchers in exploring the structure and dynamics of scientific knowledge more effectively.

## Results

### Search results

**Fig. 1:**
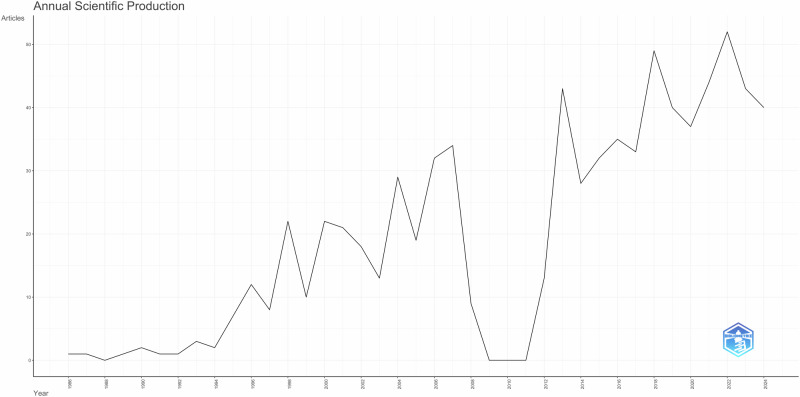
Annual scientific production. This figure illustrates the yearly trend of scientific publications over the study period.

The initial search yielded 1370 articles. Following the title and abstract screening, 1135 articles were eligible for full text screening. A total of 753 articles were included for the final analysis. It is to be noted that the annual scientific production of articles on this topic has increased considerably in the recent years (Fig. 1).

### Main collaboration and co-authorship network

**Fig. 2:**
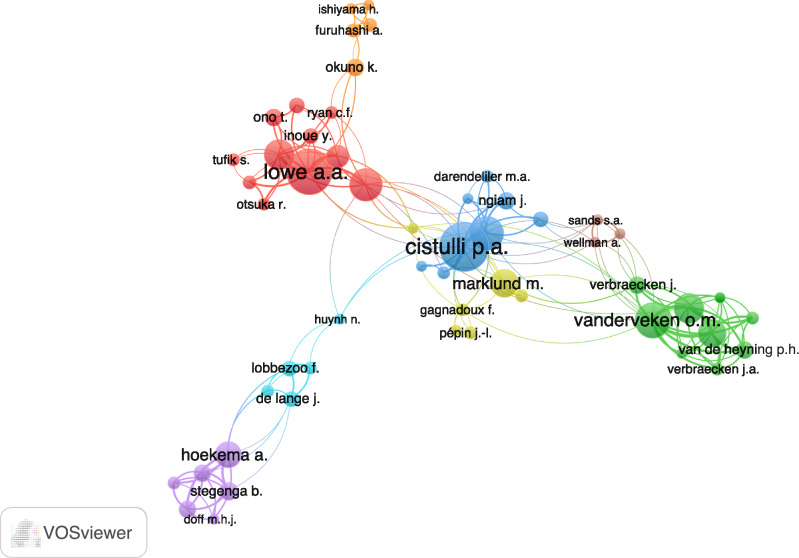
Main Collaboration and Co-authorship Network. This network diagram depicts the collaboration patterns among authors, showcasing the strength of co-authorship links through node size (representing the number of publications) and edge thickness (indicating collaboration frequency).

In terms of author impact on the subject, around 2400 researchers were identified from the 753 studies retrieved using VOS Viewer™ software. To improve the citation visualization map’s clarity, a minimum threshold of 1 citation and 5 publications was set. As a result, 53 connected authors are displayed in Fig. 2. The groups formed 8 clusters with red, blue and green clusters being the most expressive. The prominent authors in the clusters were Cistulli p.a (blue), Vanderveken o.m (green) and Lowe a.a (red). The size of the nodes in the visualization indicates the impact of each researcher’s work, based on the number of citations their publications have received. The larger the node, the greater the author’s impact and relevance in the research field. The edges of the maps show scientific collaboration between the eight clusters independently.

### Main research centers

**Fig. 3:**
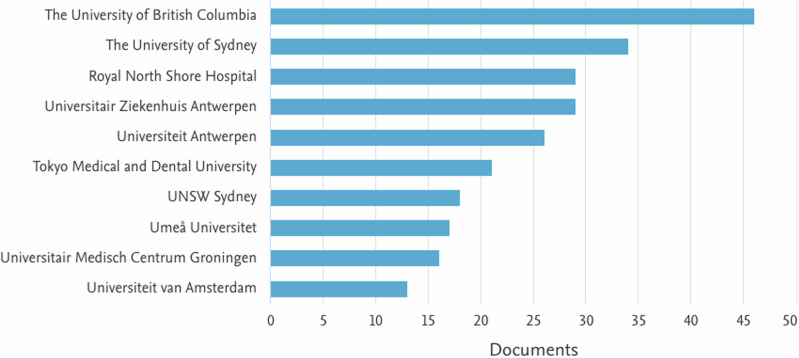
Main Research Centers. This figure presents the leading research institutions contributing to the field.

Research centers related to the topic were identified, with the three most relevant centers being: The University of British Columbia (46), The University of Sydney (34) and Royal North Shore hospital (29). The other organizations are presented in Fig. 3.

### Main countries in terms of citations and collaborations

**Fig. 4:**
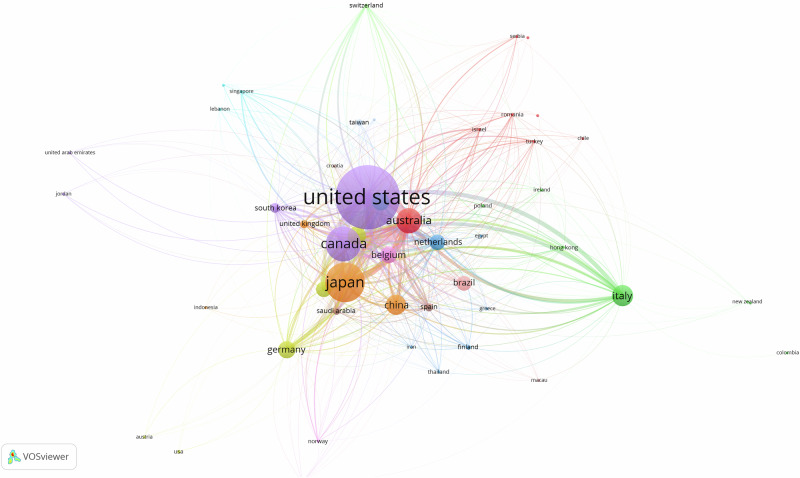
Main countries in terms of citations. This figure shows the countries with the highest citation counts and their collaboration network.

**Fig. 5:**
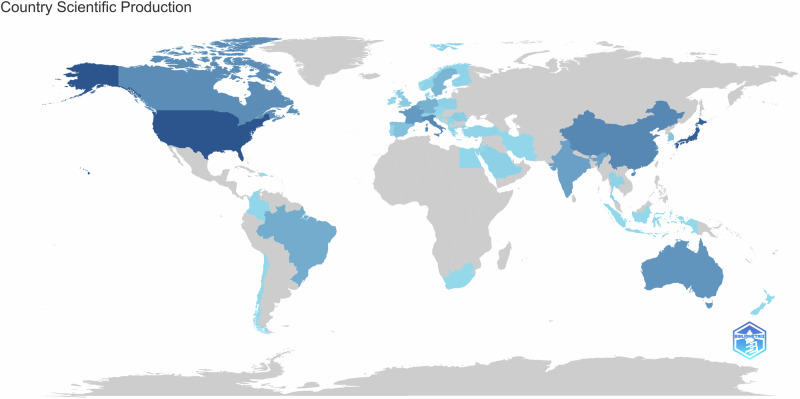
Country’s Scientific Production. This figure highlights the scientific output by country to compare publication volumes. The world map is color-coded to represent citation density, with darker shades indicating higher citation rates.

Regarding the geographical distribution of citations on the topic, when defined for 1 publication and 1 citation, 56 countries were found, as shown in Fig. 4. USA and Japan surpassed with maximal nodes, and consequently, in research on this topic, followed by Canada and Australia. There was significant link seen between many countries. To enhance ease of viewing, the world map generated by Bibliometrix software highlights the countries that contribute the most to publications on the topic (Fig. 5). The darker the shade of blue on the map, the greater the intensity of scientific collaboration on the subject, with countries displayed in navy blue being the most influential. This visualization is aligned with the network map described earlier, offering a clear representation of global research activity.

### Analysis of main keywords

**Fig. 6:**
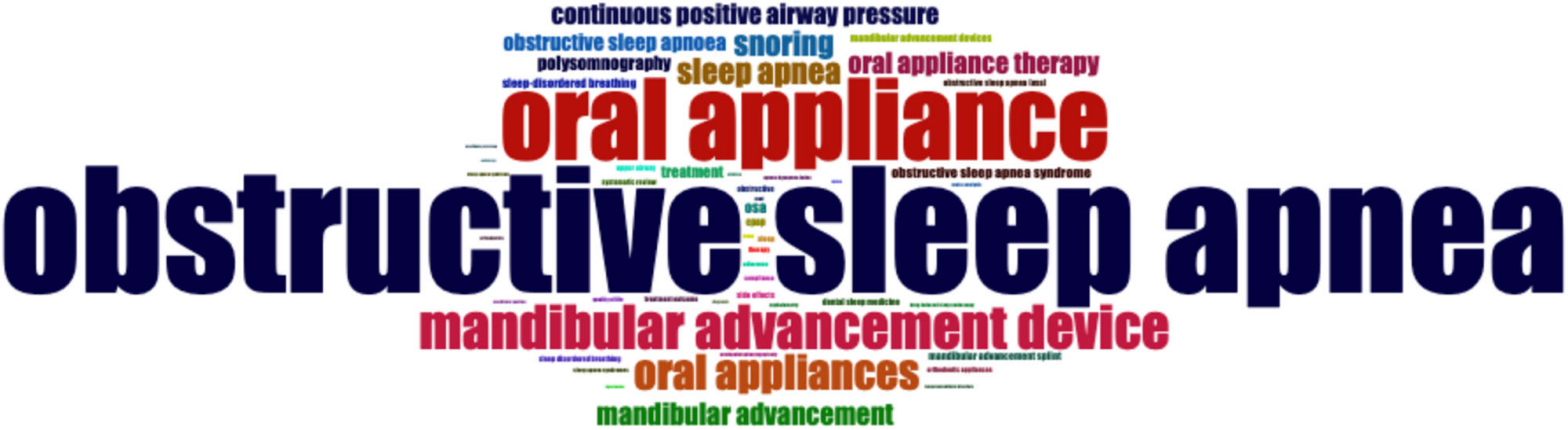
Word cloud analysis. This word cloud represents the most frequently occurring keywords in the literature. The size of each word indicates its frequency, with larger words appearing more often in the dataset.

**Fig. 7:**
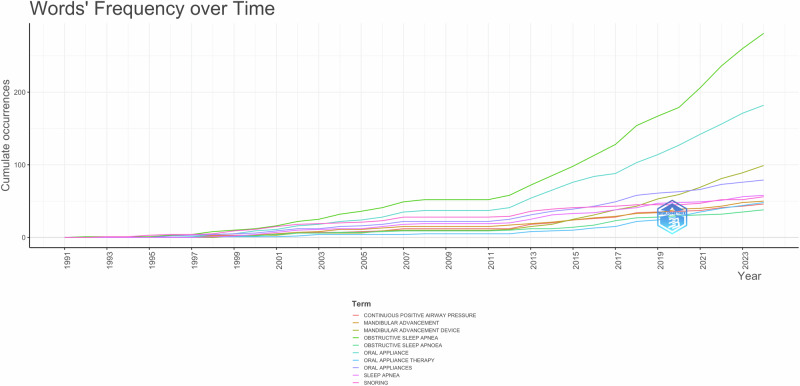
Cumulative keywords over time. This line graph tracks the cumulative frequency of key terms over time, illustrating trends in research focus.

When analyzing the co-occurrence of keywords, we chose to analyze the author’s keywords. Figure 6 enables the identification of a cloud of relevant words and acronyms. The terms obstructive sleep apnea, mandibular advancement device and oral appliance/appliances were more commonly used as keywords by authors. Words of the same color come close to the theme and have a strong connection between them, functioning as clusters. The recent trend in keywords on this topic used by authors is shown in Fig. 7. It is notable that terms like tongue retaining devices, hybrid appliances, and palatal lifting appliances are not used as keywords by authors. This suggests a potential research gap in these areas, which warrants further investigation.

### Most cited scientific journals and documents

**Fig. 8:**
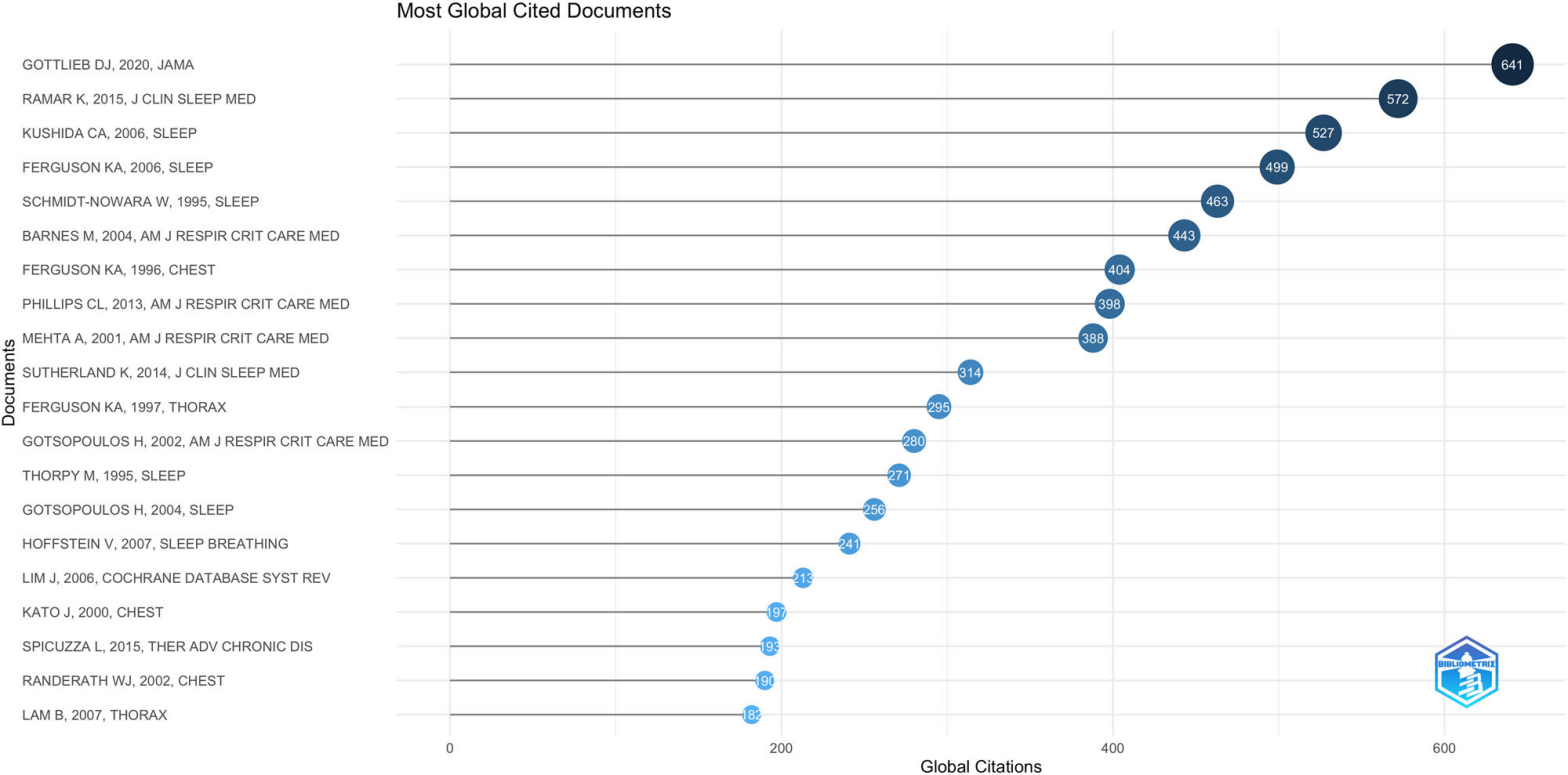
Most cited documents and journals. This figure identifies the top-cited documents and journals.

**Fig. 9:**
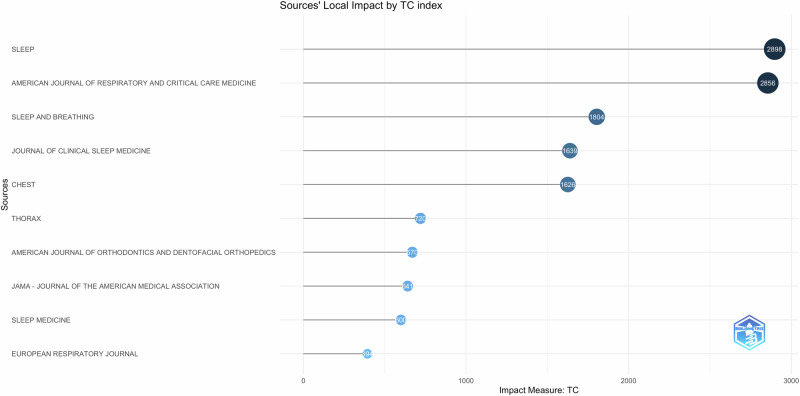
Source citations. This figure represents the impact of different sources based on citation metrics.

In Bibliometrix software, citation indicators are based on the number of citations a publication receives. The cutoff point assesses article impact and citation count, linking the number of publications to their citations. A total of 20 documents were highlighted based on citation number (Fig. 8). The 20 most-cited articles were published between 1995–2020. The number of citations varied from 10–128 per year. Of the top 10 cited documents, 4 were reviews, 2 clinical practice guidelines update, and 5 randomized clinical trials. Four out of the five clinical trials compared oral appliances to CPAP for the treatment of OSA. One randomized cross over trial tested a novel mandibular advancement appliance for the treatment of OSA. Sleep journal produced the maximum number of citations in this topic (Fig. 9).

### Most relevant authors

**Fig. 10:**
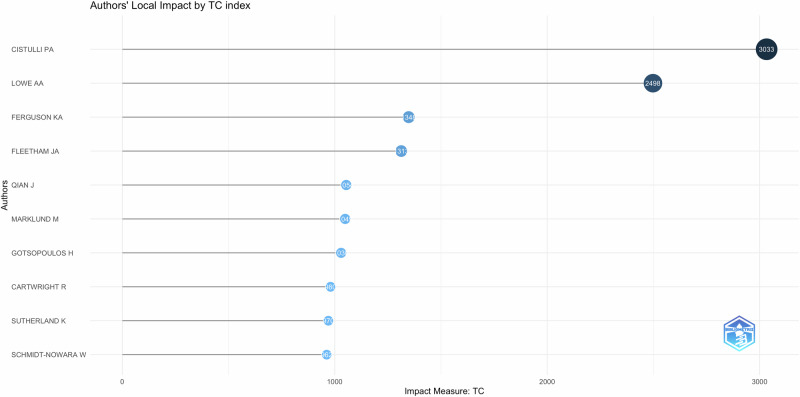
Author impact based on citations. This figure evaluates author impact through citation analysis.

**Fig. 11:**
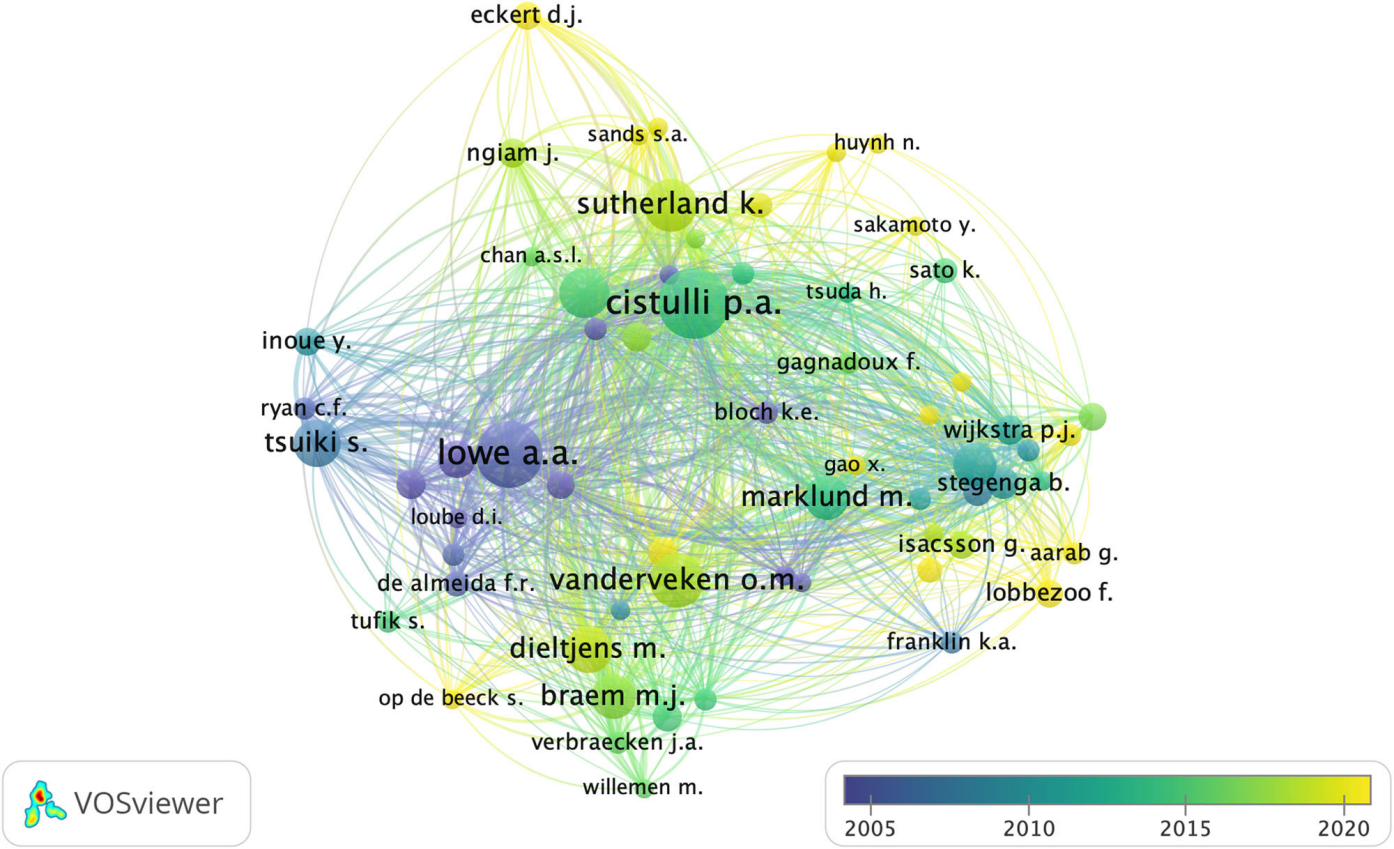
Most relevant authors and their timeline. This figure highlights the most influential authors in the field based on publication count, citation metrics and year of publication.

The most relevant authors, including the main authors and also co-authors by number of citations for this research topic, are listed in Fig. 10. Figure 11 shows the primary authors who published on the topic and the timeline of the corresponding publications. It is important to note that the timeline varied from 2005 to 2020 with an increase after 2010 when majority of the research on this topic was conducted. It is also to mention that the work done by Lowe a.a.(2005) and Cistulli p.a.(2015) is well connected with the researches conducted in 2020. This indicates that the previous research work formed a precursor for current research in this field.

## Discussion

### Narrative synthesis of top 5 cited articles

The review by Gottlieb et al. in 2020 [10] mentioned that oral appliances, particularly mandibular repositioning devices, are effective for treating mild to moderate OSA. These devices, custom-fitted to the upper and lower teeth, work by advancing the mandible, increasing upper airway volume, and reducing airway collapsibility. It also quoted a 2015 meta-analysis [11] of 34 randomized clinical trials that found that the OAs reduced the apnea-hypopnea index (AHI) by an average of 13.6 events per hour.

The 2015 clinical practice guidelines by Ramar et al. [11] highlighted substantial advancements in scientific literature, leading to updated recommendations for the treatment of OSA and snoring. A task force from the American Academy of Sleep Medicine (AASM) and the American Academy of Dental Sleep Medicine (AADSM) performed a systematic review and employed a modified GRADE process to assess the quality of the evidence. They recommended OAs for adults with primary snoring who seek treatment and suggested using custom, titratable appliances over non-custom devices for OSA. For OSA patients who cannot tolerate CPAP or prefer alternatives, OAs were recommended. Qualified dentists should oversee therapy to monitor dental side effects, and follow-up sleep testing is advised to confirm treatment efficacy. Patients should also return for periodic visits with both a dentist and a sleep physician. These guidelines aimed to enhance professional practice, improve patient outcomes, and potentially reduce healthcare costs, with future updates planned as new evidence arises.

The updated practice parameters by Kushida et al. 2006 [12] recommend OAs for patients with mild to moderate OSA who prefer them over CPAP or cannot tolerate CPAP. CPAP remains the preferred treatment for severe OSA until more evidence supports OA use. OAs should be fitted by experienced dental professionals, and follow-up polysomnography or cardiorespiratory studies are necessary to assess effectiveness and monitor symptoms. Regular follow-up visits with dental specialists are essential to ensure device efficacy and patient adherence. Further research is needed to identify factors influencing OA success and adherence.

An evidence-based review by Ferguson et al. in 2006 [13] analyzed the use of OAs for treating snoring and OSA from 1995 to the present. Out of 141 articles, 87 were included, with 15 randomized controlled trials, five of which were placebo controlled. OAs were effective in reducing apneas and hypopneas in about 52% of patients and improved sleepiness and quality of life, though neurocognitive outcomes were inconsistent. Adherence rates varied, with 77% of patients using OAs regularly after 1 year. Minor adverse effects like tooth movement were common, but major side effects were rare. While OAs are less effective than CPAP in reducing the AHI, they are often preferred by patients and compare favorably to surgical options like uvulopalatopharyngoplasty (UPPP).

The paper by Schmidt-Nowara et al. in 1995 [14], approved by the American Sleep Disorders Association, provides the foundation for the Standards of Practice in sleep medicine in North America. It reviewed 21 studies involving 320 patients treated with oral appliances for snoring and OSA. These appliances adjust the mandible and tongue to modify the upper airway, leading to consistent clinical improvements. Snoring is often eliminated, and OSA improves in most cases, with the AHI reduced from 47 to 19 on average. While 50% of patients achieved an AHI of less than 10, 40% still had elevated AHIs. Common side effects include oral discomfort, but dental complications are rare. Compliance ranges from 50% to 100%. OAs are presented as a viable alternative to CPAP, particularly for patients with simple snoring or those intolerant to CPAP therapy.

The randomized controlled crossover trial by Branes et al. in 2004 [15], involving 114 patients with mild to moderate OSA (AHI 5–30) compared the effects of nasal CPAP, a mandibular advancement splint, and a placebo. Both CPAP and the splint improved sleep outcomes, though CPAP had a greater effect. Quality of life, symptoms, and subjective sleepiness improved similarly with both treatments, but neuropsychological improvements were comparable to the placebo. Some aspects of nocturnal blood pressure improved with the splint but not with CPAP. Despite the effectiveness of both treatments, CPAP’s lower usage and the splint’s reduced therapeutic effect may have limited improvements in neurobehavioral function.

### Identified research gaps in this bibliometric analysis

OAs, particularly mandibular repositioning devices, are effective in treating mild, moderate and severe OSA, with evidence from multiple studies and clinical guidelines supporting their use as an alternative to CPAP therapy, especially in patients who prefer or cannot tolerate CPAP [16, 17]. While the majority of highly cited studies have not addressed the use of MAD in severe OSA, recent research is beginning to emerge, highlighting their potential effectiveness in this patient population. These newer studies are gradually providing evidence to support MAD as a viable treatment option even for those with severe OSA. Despite the established efficacy of OAs in mild, moderate and severe OSA’s, several research gaps have been identified in the present bibliometric analysis. One key area is the variability in patient response, with some individuals achieving significant AHI reduction while others see limited improvement. More research is needed to identify the predictors of successful treatment outcomes, including the role of craniofacial anatomy and airway characteristics. Long-term studies on adherence and the durability of these appliances are limited, particularly regarding side effects like tooth movement or temporomandibular joint disorders. Another gap lies in comparing different OAs to newer CPAP alternatives and surgical interventions in diverse patient populations, including those with severe OSA. Additionally, there is limited research on the impact of OAs on cardiovascular outcomes, neurocognitive function, and overall quality of life beyond sleepiness reduction. Future studies should also explore more standardized protocols for follow-up care and sleep testing to optimize treatment efficacy and address complications.

### Study limitation

While Scopus includes a broad range of sources, including books, book chapters, and online clinical decision tools, these were intentionally excluded from this study. This decision was made to prioritize peer-reviewed journal articles, which undergo a rigorous review process to ensure consistency, quality, and reliability. In contrast, books and online resources often lack standardized peer-review mechanisms and may vary significantly in the depth and accuracy of the information presented. By focusing exclusively on peer-reviewed articles, the study aimed to ensure a robust and reliable evidence base, though this approach may have limited the inclusion of potentially valuable insights from non-journal sources.

Another limitation of this study is the potential issue within Scopus related to authors having multiple profiles. This often occurs due to variations in name spelling, changes in institutional affiliations, or inconsistencies in author details over time. Such discrepancies can result in the fragmentation of an author’s publication record, leading to overestimation or underestimation of their contributions, skewed citation counts, and distorted co-authorship networks. These issues may affect the identification of influential researchers, the calculation of h-index values, and the overall mapping of research collaborations, thereby impacting the accuracy and reliability of the bibliometric analysis.

## Conclusion

Bibliometric analysis offers a strategic approach to identifying research gaps in the use of oral appliances for sleep apnea. By examining publication trends, citation patterns, and key contributors in this field, it reveals under-researched areas. This method also highlights research gaps, as well as the need for more robust data such as on patient adherence and quality of life improvements. By pinpointing these research gaps, bibliometric analysis helps direct future investigations and collaborations, ensuring that critical unanswered questions about oral appliances in sleep apnea are effectively addressed.

## Data Availability

The datasets used and/or analyzed during the current study are available from the corresponding author on reasonable request.
